# Neuroimaging for neurovascular complications of traumatic brain injury

**DOI:** 10.1186/s13054-025-05586-2

**Published:** 2025-08-18

**Authors:** Laura Saitta, Martina Resaz, Chiara Robba, Giacomo Rebella, Claudia Rolla Bigliani, Giancarlo Salsano, Bruno Del Sette, Nicola Mavilio, Francesco Pasetti, Nicolò Patroniti, Lucio Castellan, Luca Roccatagliata

**Affiliations:** 1 U.O. Neuroradiologia, Ospedale Policlinico San Martino, IRCCS for Oncology and Neuroscience, Genoa, Italy; 2 U.O. Neuroradiologia, Istituto Giannina Gaslini IRCCS, Genoa, Italy; 3 Intensive Care Unit, Ospedale Policlinico San Martino, IRCCS for Oncology and Neuroscience, Genoa, Italy; 4 Dipartimento di Scienze Chirurgiche Integrate e Diagnostiche, University of Genoa, Genoa, Italy; 5 U.O. Radiologia, Istituto Giannina Gaslini IRCCS, Genoa, Italy; 6 Dipartimento di Scienze della Salute, University of Genoa, Genoa, Italy

**Keywords:** Traumatic brain injury, Neuroimaging, Neurocritical care, Neurovascular complications

## Abstract

**Background:**

Traumatic brain injury typically causes extra-axial and/or intra-axial bleeding including subarachnoid hemorrhage, intraparenchymal hemorrhage, subdural hematomas and epidural hematomas. Less commonly, trauma can cause cerebrovascular complications, which involve either the arterial or the venous side. Because of the rarity of these pathological conditions, guidelines and recommendations for their management are still controversial.

**Main body:**

The objective of this work is to describe the possible cerebrovascular complications of critically ill traumatic brain injured patients and to understand the most common underlying mechanisms and radiological features as well as their management. A variety of pathological entities will be addressed, such as post-traumatic aneurysms, carotid-cavernous fistula, arterial occlusion, arterial dissection (in potential association with brain ischemia), as well as arterial rupture/avulsion and post-traumatic venous thrombosis. Neurovascular complications of head trauma vary depending on the traumatic mechanism, on the site of impact and on the osseous structures involved. Early diagnosis is mostly based on Computed Tomography/Computed Tomography Angiography (CT/CTA) whose findings help guide patient management by detecting vascular lesions potentially leading to neurological deterioration. Magnetic resonance imaging may be useful in selected cases. Today Digital Subtraction Angiography (DSA) is mostly a diagnostic problem-solving tool when CTA findings are equivocal but advanced endovascular interventional techniques have improved the therapeutic possibilities in post-traumatic vascular complications.

**Conclusions:**

Neurovascular complications are not common after head trauma but should not be overlooked because they might lead to severe and life-threatening consequences. Early diagnosis, and a multidisciplinary collaboration including neuroradiologists, neurosurgeons and neurointensivists is fundamental in order to prevent and minimize secondary brain damage in this population.

## Background

**Fig. 1:**
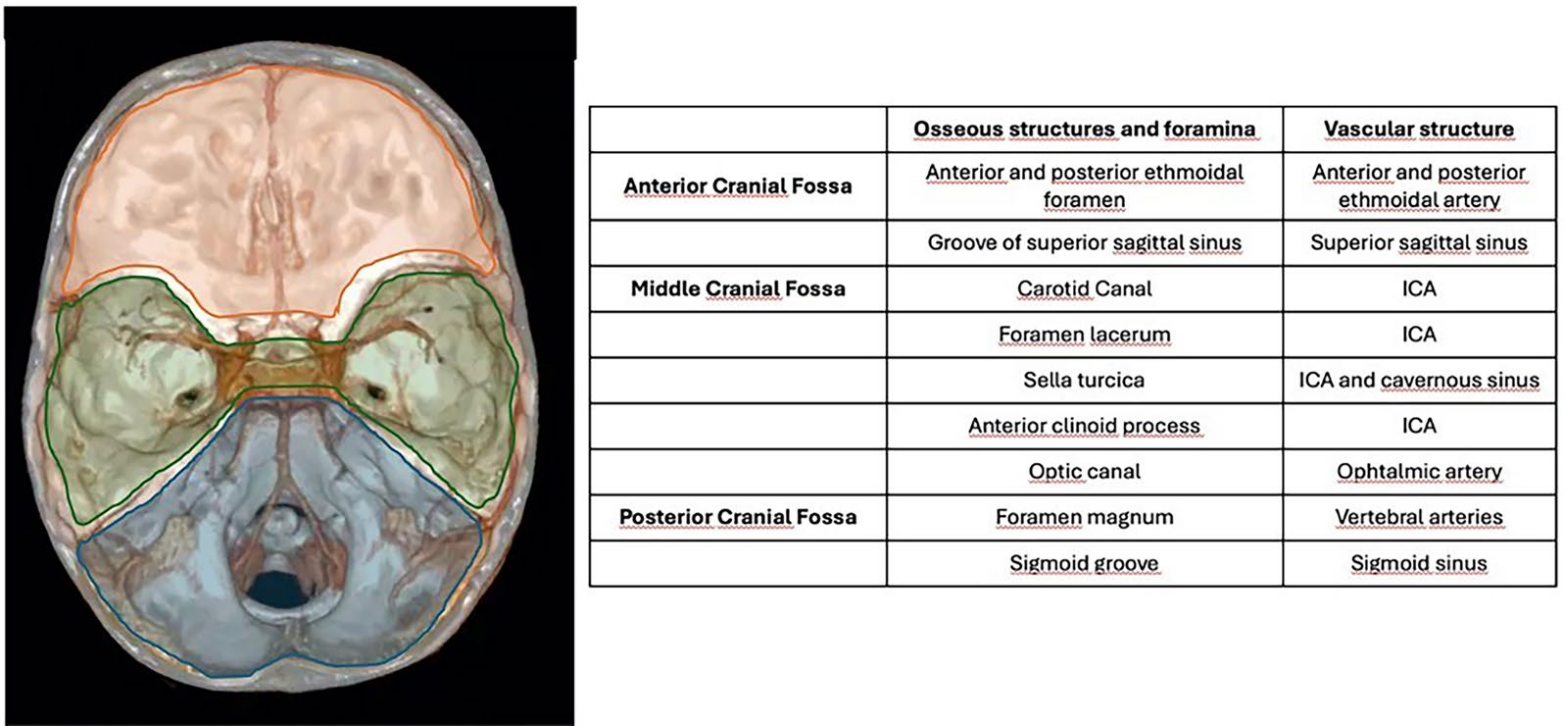
Anatomy of the osseous structures and foramina observed in the anterior, middle and posterior cranial fossa, with respect to the vascular structures which could possibly be injured when involved in TBI

Neurovascular complications of traumatic brain injury (TBI) have an incidence of 1–9% of cases according to recently published evidence [1, 2]. The spectrum of post-traumatic neurovascular injuries encompasses lesions involving the arterial and venous vasculature, including arterial dissection, traumatic aneurysms, arterial transection, artero-venous fistula (AVF) and venous thrombosis. The pathogenesis depends on the traumatic mechanism, on the site of impact and on the osseous structures involved (Fig. 1).

The most common mechanism causing brain or neck vascular injury is blunt trauma, while penetrating trauma is less frequent [3]. Blunt Cerebrovascular injury (BCVI), related to motor vehicle accidents, assaults or contact sports’ injuries, can result from sudden acceleration-deceleration mechanism, hyperextension, hyperflexion or rotational trauma of the neck [4]. In penetrating trauma low-velocity mechanisms (e.g. stab wounds) cause vascular damage along the path of penetration, while high-velocity mechanisms (e.g. gunshot wounds) may cause injuries which extend beyond the penetration path due to the dissipation of energy [2, 5].In general penetrating TBI has a very high mortality rate with approximately 66–90% of patients dying before reaching the hospital [6].

Vascular injuries are a common complication in penetrating TBI and include traumatic aneurysms formation occurring at a rate of 2–33%, and more commonly involving distal branches of middle cerebral arteries and anterior cerebral artery, with respect to BCVI. Arterial injuries after penetrating TBI are predicted by the entry wound over the fronto-basal and temporal regions, biemispheric trajectory and a trajectory adjacent to the anatomical location of the circle of Willis, with evidence of subarachnoid and/or intraventricular hemorrhage at admission head CT scan [7]. Only about 37% of patients have clear symptoms of brain ischemia at first presentation in the emergency room after TBI [8], but BCVI patients with non-treated neurovascular injuries have a risk of ischemic stroke of about 30–40% when involving the carotid artery and of about 10–15% for vertebral artery [9]. Early identification of cerebrovascular injury through implementation of proper radiological screening criteria and appropriate management may therefore improve patients’ outcomes, especially by reducing the risk of secondary ischemic stroke after TBI [10]. Also, other adverse outcomes beyond ischemic stroke, such as hemorrhage from traumatic aneurysms, significant neurological deficits from AV fistulas, may be prevented. In critically ill sedated or neurologically compromised patients, not only neuroimaging and screening protocols are fundamental to achieve early diagnosis and minimize poor outcomes, but also increased awareness of ICU medical and non medical staff, optimization of medical management, and earlier treatment strategies, by applying best practice guidelines [11]. 

Neuroimaging can therefore play an important role in this context, especially for critically ill TBI patients when the neurological status is poor or sedation is required to manage intracranial hypertension or to help synchrony with the ventilator and cannot be clinically assessed.

### Type of neuroimaging to use

When a neurovascular complication is suspected, CT-Angiography (CTA) represents the imaging technique of choice [11], although digital subtraction angiography (DSA) is still reported as the gold standard for BCVI detection in the setting of trauma patients at risk of neurovascular damage [4].

CTA is widely available and can easily and rapidly complement non contrast CT studies in the emergency setting, enabling significantly faster diagnosis of BCVI when compared to DSA [12]. Even if single institution studies have reported high sensitivity and specificity of CTA in detecting BCVI (up to 98% and 100%, respectively [12]) there are large differences among different studies in the diagnostic performance of this tool. Meta-analyses of studies comparing CTA by using CT scanners up to 64-detector rows and DSA report a pooled sensitivity of 66%−64% and specificity of 97% −95% for CTA [13, 14], suggesting limited performance in excluding the presence of neurovascular complications. The relatively low sensitivity has led to concerns regarding potential under-treatment in patients with a false negative CTA study. In fact, CTA is more likely to correctly identify more severe grades of vascular damage. Thus, missed cases are more commonly low-grade BCVI where there is not always established evidence for clinical management with anticoagulation or anti-aggregation [14].

Interestingly, other studies using DSA to confirm positive CTA findings have reported a CTA high false positive rate in low grade vascular injuries with a positive predictive value of only 30% [15]. These variations in the diagnostic performance among different studies may be related to various factors, such as dissimilarities in training and experience, different thresholds for reporting mild injuries and numerous technical aspects related to image acquisition. In addition, future studies are needed to assess the diagnostic accuracy of CTA acquired with a higher number of detector rows (e.g. 256 detector rows) [14, 16].

The use of DSA in ambiguous CTA findings of low-grade vascular injury might not be warranted and confirmation can be achieved with a follow up CTA without compromising clinical management [11, 17]. Indeed, from a clinical perspective, most low-grade BCVI might not require anticoagulant or antiplatelet therapies, as the risk of hemorrhage outweighs the risk of ischemia, especially in the acute phase.

However, all in it, these findings shed the light on a different scenario where trauma patients with multiple systems injuries and at risk of hemorrhagic complications might be inappropriately treated with anticoagulant or antiplatelet therapies [18].

In these instances, DSA might be considered to confirm CTA findings.

Finally, magnetic resonance imaging (MRI) and MR-Angiography (MRA) are less frequently acquired in the emergency setting due to the concern for metallic foreign bodies related to trauma, longer acquisition times and sensitivity to motion artifacts. Despite these limitations, MRI can improve diagnosis of BCVI. For instance, in low-grade lesions with equivocal BCVI on CTA, MRI with vessel wall imaging allowing for direct visualization of arterial wall might improve diagnostic accuracy of BCVI [19].

### When to image

The increasing adoption of screening criteria and a more strict neurological evaluation especially in critical care has improved the detection of BCVI [9], with a concomitant reduction of BCVI-related ischemic stroke and mortality over the last three decades. In particular, the occurrence of stroke has consistently decreased over the years from 37% to about 4,8% of patients with BCVI [20, 21].

A collaborative approach of neurointensivists and neuroradiologists should help in recognizing the instances which require to proceed with CTA in a trauma patient after a plain CT head.

The European Society of Emergency Radiology guidelines on radiological polytrauma imaging state that the neck region should be included in the whole body tomography scan with intravenous contrast medium in such a way that the neck arteries and brain base arteries are well opacified [22].

According to the guidelines of the American College of Radiology in penetrating neck injuries with clinical soft injury signs, and patients with hard signs of injury who do not require immediate surgery, CTA of the neck is the imaging of choice to evaluate the extent of injury [23].

Screening protocols for blunt cerebrovascular injuries were developed for the first time at the end of 1990 s and focused on injury mechanisms, physical symptoms and fractures type that predicted BCVI [24]. Screening criteria were later modified detailing the type of cervical spine fracture and other injury patterns that were associated with BCVI [25]. A further revision of screening criteria was suggested in 2011 [26] and then included in the modified Denver criteria (DC) in their expanded version [9] (Table 1). These are the most used criteria for screening blunt cerebrovascular injuries with CTA and include clinical and radiological factors as well as risk factors related to the dynamic of trauma. A more recent systematic review and meta-analysis on the use of CTA for screening identified an association with BCVI only with fractures in the C1-C2 region, with two-level fractures, subluxations/dislocations and transverse foramen fractures [27].

**Table 1:**
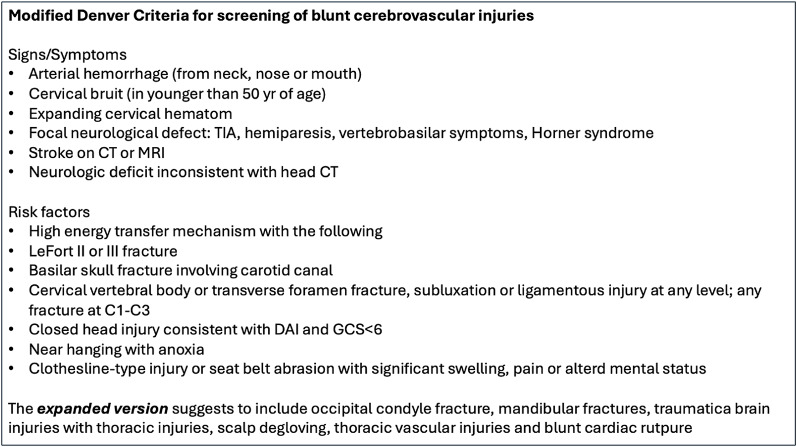
Modified Denver criteria

The Boston criteria [28] (Table 2) are based on the Denver criteria, but they are divided into two categories depending on whether the indication for CTA acquisition needs to be immediate or can be planned within 24–48 h from presentation. The use of the Denver and Boston criteria can help the intensivists to ensure that proper neuroradiological work-up has been obtained at the correct time.

**Table 2:**
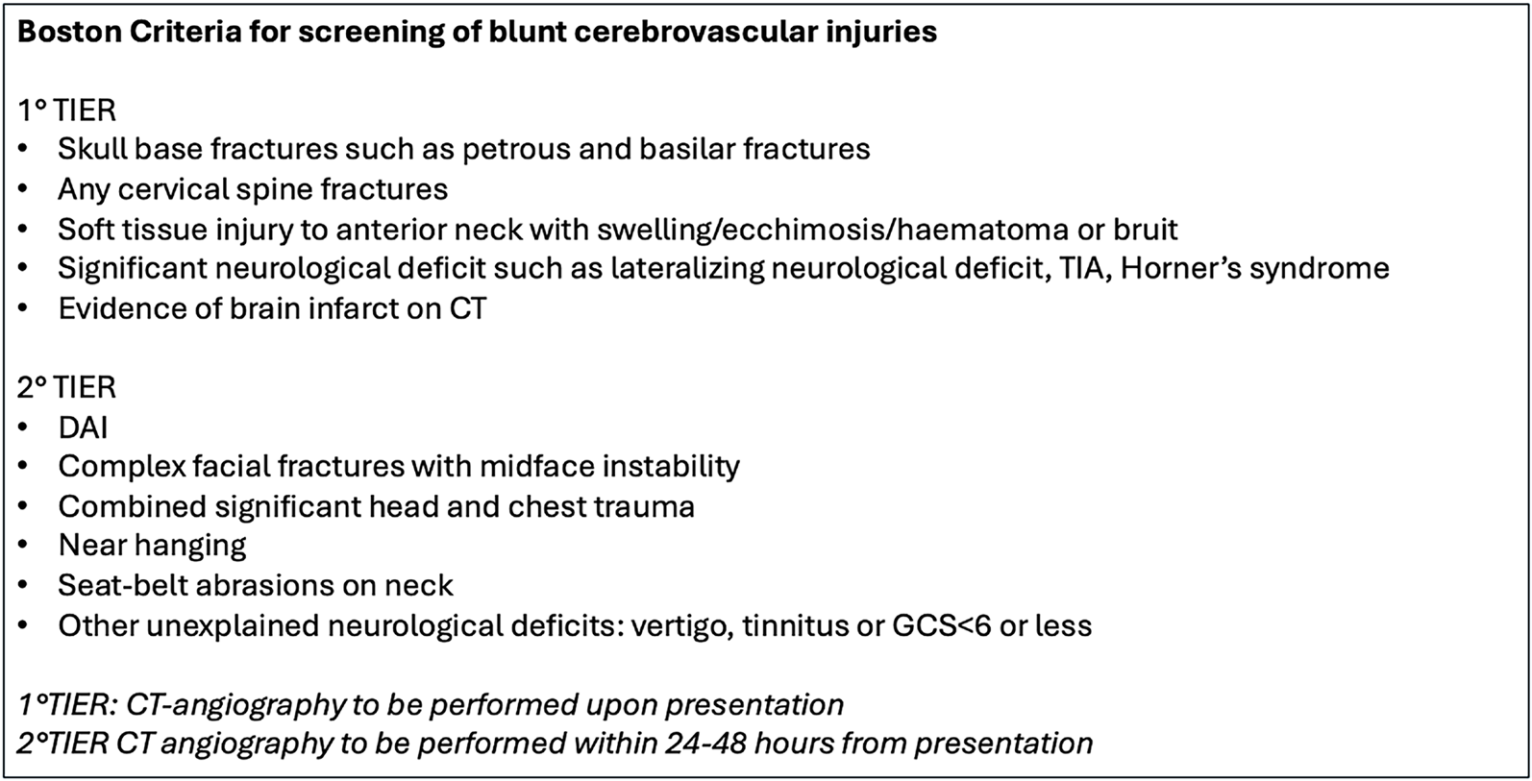
Boston criteria

**Fig. 2:**
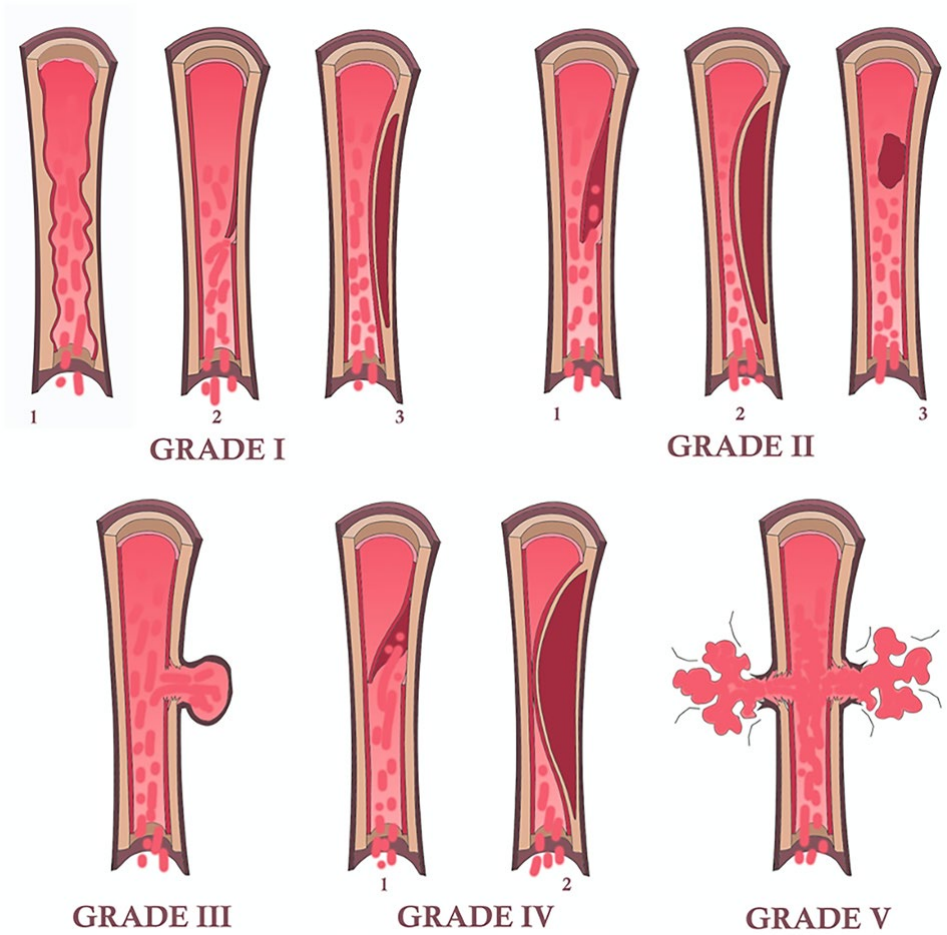
Schematic drawing of the five grades of vascular injury according to the Denver scale. Grade I may present as irregularity of the vessel wall [1], subtle intimal flap [2] or intramural hematoma with < 25% stenosis; grade II may present as frank intimal flap [1], intramural hematoma with > 25% stenosis [2] or intraluminal thrombus [3]; grade III is represented by the presence of a pseudoaneurysm; grade IV occurs in case of arterial occlusion by a complete intimal flap [1] or by an intramural hematoma [2]; grade V is represented by arterial transection

The grading of vascular injury can be assessed by using the Denver scale, which should not be confused with the DC. This scale classifies the features of vascular injury in 5 grades (I-V), with increasing risk of brain ischemia in relation to higher grades of arterial damage (carotid arteries or vertebral arteries) [29, 30]. The Denver scale was primarily based on DSA findings and later adapted to CTA (Table 3). Figure 2 shows a schematic drawing of the five grades of the Denver scale.

**Table 3:** Denver scale: the features of the 5 grades (I-V) of vascular injury are shown, with increasing risk of brain ischemia in relation to higher grades of arterial damage

Grade	Denver grading system	CT-Angiography findings
**I**	Irregularity of vessel wall Dissection or intramural hematoma with < 25%narrowing	Nonstenotic luminal irregularity Intimal flap or wall thickening with < 25% stenosis
**II**	Intraluminal thrombus Dissection or intramural hematoma with > 25% narrowing	Luminal hypodensity Intimal flap or wall thickening with > 25% stenosis
**III**	Pseudoaneurysm	Eccentric contrast-filled outpouching limited by periarterial tissue
**IV**	Occlusion	Lack of any intraluminal enhancement Carotid occlusions: abrupt or tapered Vertebral occlusion: usually abrupt
**V**	Transection	Irregular extravascular collection of contrast, not limited by periarterial tissue Increases in density on delayed images, if obtained

Despite the development of dedicated screening criteria for blunt cerebrovascular injury with CTA in patients with trauma, there is still no universal agreement on which patients should be screened and some institutions foster a more liberal use of CTA in trauma patients. Different studies showed that BCVI can be missed in approximately 20 to 30% of cases using the DC or the eDC [31–33]. and thus consider a universal approach for screening. Drawbacks of this approach include radiation exposure and increased costs due to the higher number of CT requisitions. Also, some authors underline that with a more liberal use of CTA comes the risk of a high number of indeterminate findings, ultimately revealed as false positive, or subtle vessel contours anomalies related to vasospasm which may lead to unnecessary and potentially dangerous treatments in trauma patients [34] and to the identification of low grade injuries with little clinical relevance [14].

#### Arterial Dissection/occlusion

**Fig. 3:**
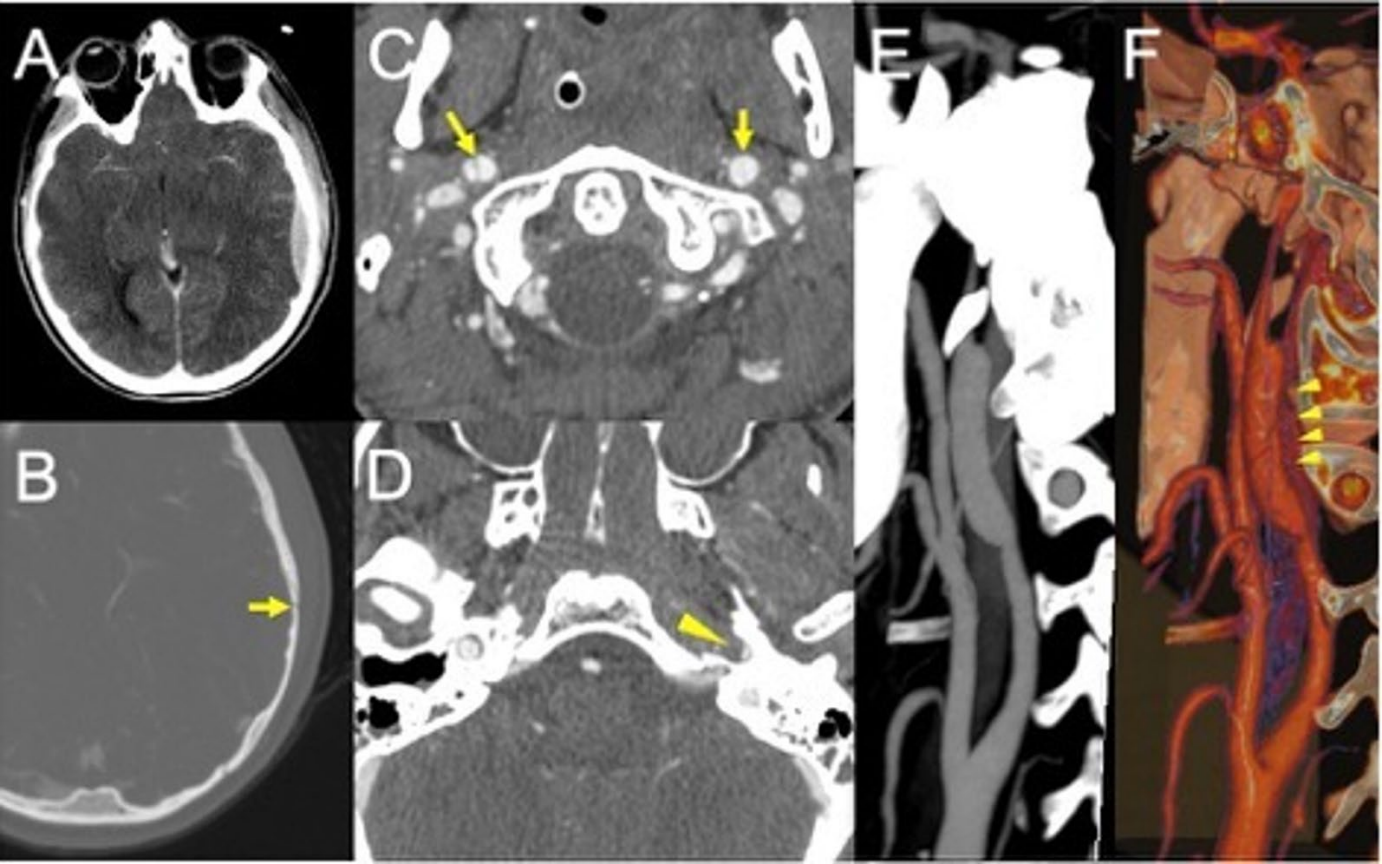
Post-traumatic bilateral internal carotid dissection and left carotid fusiform dilatation in an adolescent patient following a motor vehicle accident. CT scan shows a parieto-temporal epidural hemorrhage (**A**) in association with a skull fracture at the same level (**B**, arrow). CTA demonstrates a bilateral double lumen sign (**C**, arrows), consistent with the presence of intimal flap and a distinct narrowing of the left vertical petrous carotid (**D**, arrowhead). Maximum intensity projection (**E**) and 3D volume rendering (**F**, arrowheads) show the presence of a dilatation of the left carotid artery (consistent with a pseudoaneurysm due to a tear of the intima and hemorrhage into media and adventitia layers)

In blunt trauma, craniocervical arterial dissection occurs in about 1–2% of patients, with higher risk in the presence of skull base fractures, facial fractures (e.g. Lefort fractures) or traumatic brain injury [35] (Fig. 3). Major thoracic traumas increase the risk of carotid arteries dissection, whereas spinal traumas with cervical fractures or spinal cord lesions increase the risk of vertebral arteries (VA) dissection [36].

Dissections can involve the arterial segments between anchored points, that are the most vulnerable by motion and stretching or by contact with adjacent rigid bony structures (37), or involve segments adjacent to a bone fracture, as depicted in the Boston criteria.

**Fig. 4:**
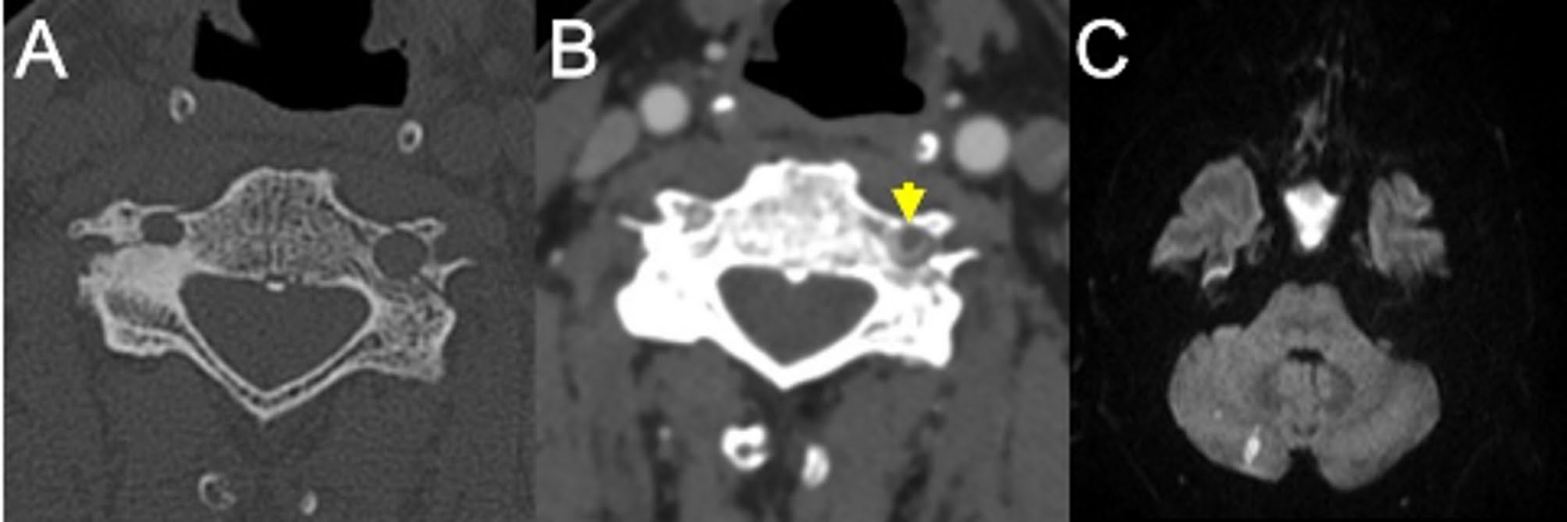
Post-traumatic cervical CT, CTA and MRI of a patient in the sixth decade of life involved in a car accident. Non-enhanced CT scan shows bilateral fractures of the lateral masses of a cervical vertebra (**A**), with involvement of the transverse foramina. CTA (**B**) shows partial lack of opacification of the left vertebral artery, consistent with post-traumatic arterial dissection (arrow) (Denver grade II). The MRI (**C**) shows foci of restricted diffusion in the diffusion weighted sequence in the right cerebellum, consistent with acute ischemic lesions of embolic origin from the left vertebral artery

**Fig. 5:**
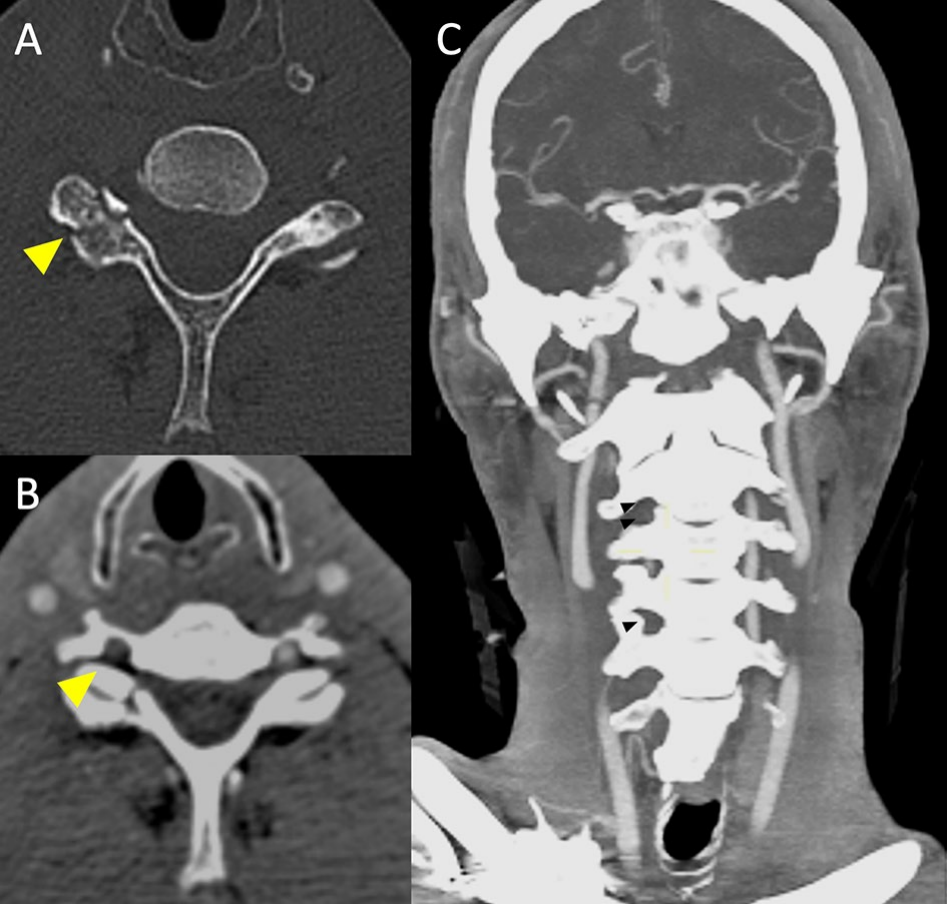
Basal CT scan of a patient in the early fifth decade of life following a motor vehicle accident. Non-enhanced CT scan (**A**) demonstrates a fracture of the right superior articular process of the C6 vertebra (yellow arrowhead). CTA (**B**, **C**) shows lack of opacification of the right vertebral artery in the transverse foramen (**B**, yellow arrowhead), starting from the distal V1 segment (not shown), continuing along part of the V2 segment (**C**, black arrowheads) and V3 segment and ending at the proximal V4 segment (not shown), consistent with arterial dissection with subsequent occlusion of the vessel (Denver Grade IV)

**Fig. 6:**
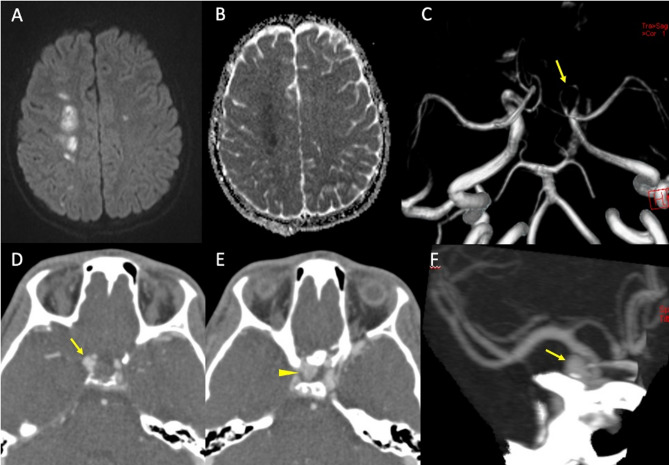
Blunt trauma in a patient at the beginning of the third decade of life involved in a motor vehicle accident. MRI (top row) shows areas of restricted diffusion (**A**: diffusion weighted imaging sequence and **B**: apparent diffusion coefficient map) in the centrum semiovale of the right cerebral hemisphere, consistent with acute ischemia. MRA (**C**) demonstrates distinct narrowing of the right intracranial carotid artery (arrow) involving the cavernous and supraclinoid segments, consistent with post-traumatic dissection. Follow-up CTA one month from presentation (**D**-**F**) shows the appearance of a bilobated post-traumatic aneurysm (Denver grade III) of the right internal carotid artery, with a component arising from the supraclinoid tract and directed supero-laterally (**D**, arrow) and a component arising from the cavernous portion and directed medially in the sellar region (**E**, arrowhead). Maximum intensity projection reconstruction (**F**) more clearly depicts the supraclinoid portion of the aneurysm (arrow)

The VA dissections commonly involve the V1 segment, before the artery enters the foramina transversaria (the V2 segment) and the V3 segment at the atlas loop following arterial stretching [37]. For ICA dissections, the pharyngeal portion is a very common location. VAs injuries related to fractures, are most common in the pars transversaria in association with fracture of the transverse processes (Fig. 4), while ICAs dissections can be related to fractures involving the carotid canal. Understanding the type of involvement of the three arterial layers (intima, media and adventitia) is crucial to comprehend the possible consequences of an arterial dissection, and eventually balance the risk and benefits for antiplatelets therapy versus risk of bleeding. A dissection typically results from a tear of the intimal layer with formation of an intimal flap and penetration of blood in the media layer with secondary formation of an intramural hematoma or from the primary formation of an intramural hematoma in the media layer, by direct bleeding from the vasa vasorum. Intramural hematomas tend to extend cranially in the direction of the bloodstream and may enlarge and cause narrowing or occlusion of the vessel lumen (Fig. 5) [20]. As it grows, the intramural hematoma, may increase the external diameter of the vessel and may reach and dilate the adventitia layer, with formation of a pseudoaneurysm [38]. Hence, the risk of cerebral ischemia associated with arterial dissection is related to different mechanisms. A first mechanism is the occurrence of thromboembolism from platelets aggregates formation at the level of the intimal tear or with emboli originating within a pseudoaneurysm connected to the intimal tear. A second cause results from arterial lumen narrowing/occlusion caused by enlargement of the intramural hematoma with a consequent stroke of hemodynamic nature (Fig. 6) [35].

Intradural extension of cervical dissections, more common in VA dissection than in ICA dissection, increases the risk of stroke and cerebral ischemia, reported in up to 78% of patients with mechanisms that also include occlusion of perforating branches originating from the dissected parent artery [39].

Intracranial dissections can also manifest with subarachnoid hemorrhage (SAH), reported in 50–60% of patients and caused by a transmural arterial involvement. Intradural arteries are constituted by a very thin adventitia, by a scarcity of elastic fibers in the media layer, with no external lamina elastica and a thicker internal lamina elastica [40] that makes them susceptible to transection. A careful balance considering the risk of thrombosis/embolism and the presence of active or intracranial bleeding should guide clinical management for the initiation of antiplatelets therapy.

**Fig. 7:**
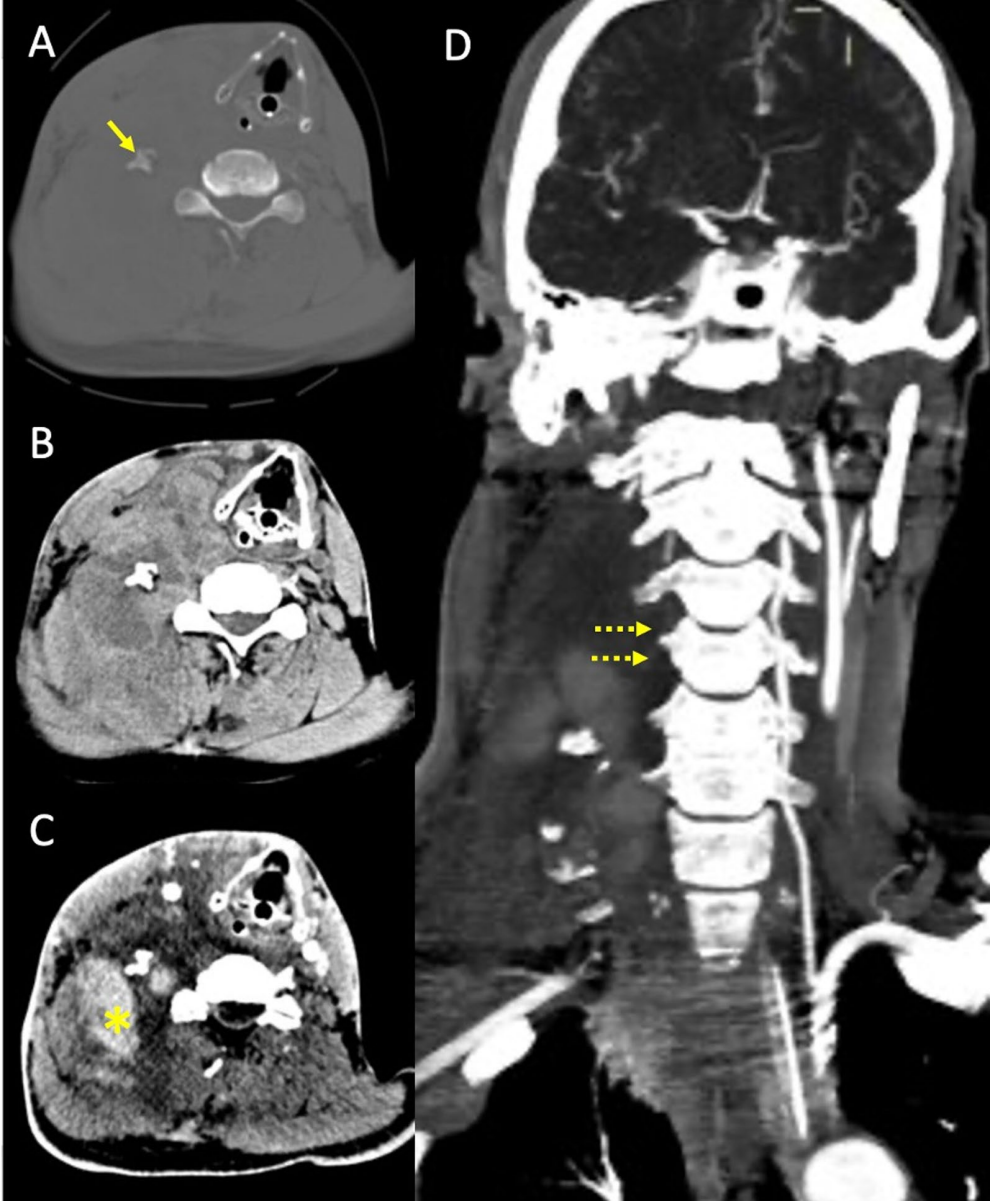
Right vertebral artery rupture after motor vehicle collision in a patient in the seventh decade of life. Non-enhanced CT scan (**A **and **B**) at the level of C5 shows fracture and lateral dislocation of the right transverse process (arrow), with ipsilateral distinct collection in the soft tissues of the neck (**B**). CTA (**C**) shows contrast extravasation in the right lateral cervical soft tissues, consistent with acute hematoma (asterisk), with lack of opacification of the right vertebral artery, best seen in the maximum intensity projection reconstruction (**D**, dotted arrows), most consistent with vertebral artery avulsion

Traditionally, the conventional imaging method to diagnose arterial dissection has been DSA [39, 41]. Today, arterial dissections are typically diagnosed with CT/CTA or MR/MRA. When evaluating traumatized patients in an emergency setting, CTA is the method of choice to diagnose arterial dissection. CTA may show different findings including the presence of an intimal flap, which may appear as a double lumen sign, irregularity of the vessel lumen with different degrees of stenosis, which may be associated with thickening of the vessel wall related to intramural hematoma. Intramural hematoma is typically less conspicuous on CT than MRI. Other CTA findings include the presence of an external outpouching consistent with pseudoaneurysm, absence of intravascular contrast enhancement with a tapered or abrupt interruption of arterial opacification; in case of arterial transections there can be irregular extravascular collection of contrast (Fig. 7).

Non-contrast CT is usually not diagnostic but may show subtle changes such as an enlarged external arterial diameter and hyperdense intramural thrombus; commonly though there can be limited difference in contrast between the thrombosed false lumen and the adjacent tissues, limiting the assessment on the presence and extension of the dissection.

**Fig. 8:**
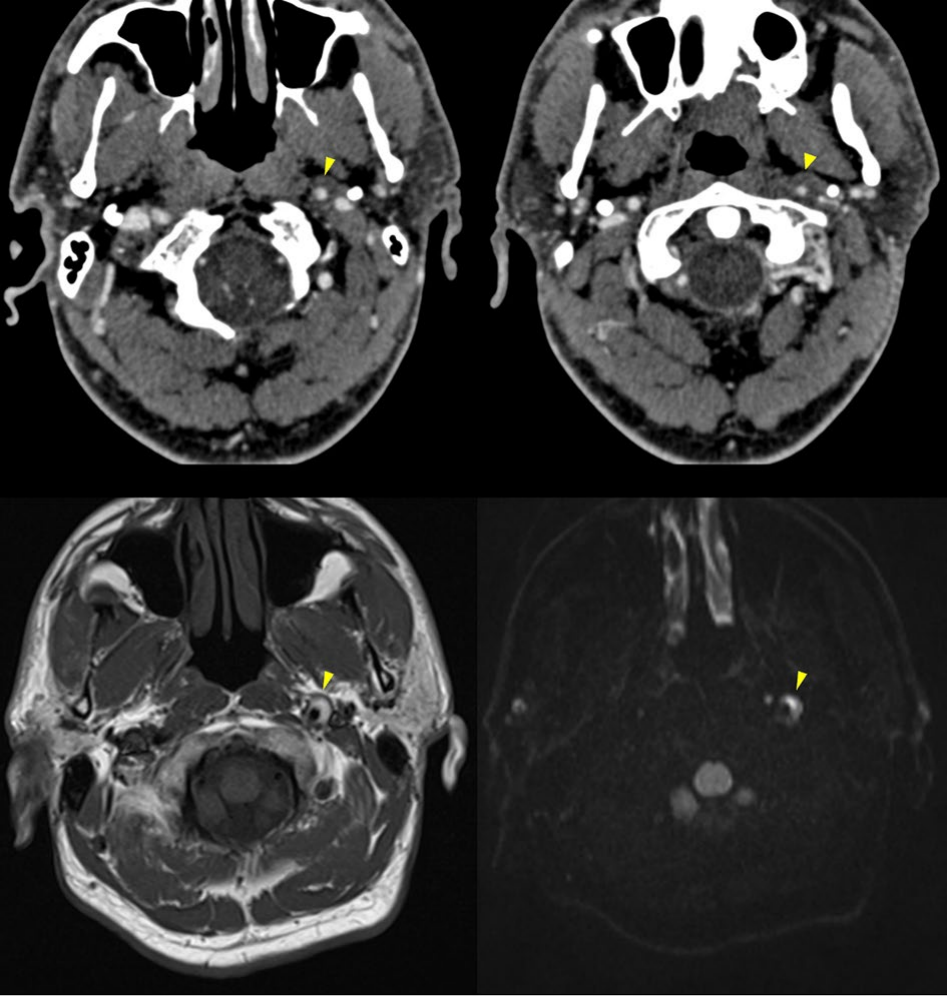
CTA and MRI findings of arterial dissection in the presence of a mural hematoma. CTA (top row) shows a slight lumen reduction of the distal cervical segment of the left internal carotid artery, where an intramural hematoma is also suspected, showing isodensity with the surrounding soft tissues (arrowheads). MRI (bottom row) at the same level more clearly depicts a “crescent sign” consistent with a subacute intramural hematoma on T1-weighted (bottom left) and on DWI (bottom right) sequences (arrowheads)

MR imaging is more sensitive for the detection of an intramural hematoma, which can typically be detected as a crescent eccentric to the dissected arterial wall. The conspicuity of this so-called crescent sign depends on the age of blood. In the subacute stage of thrombus the intramural hematoma is best seen on T1-weighted sequences with fat-suppression as a T1-hyperintense crescent reflecting the presence of intracellular or extracellular methemoglobin and is possibly associated with a luminal narrowing [42]. Intramural hematomas may appear isointense to the surrounding tissues in T1-weighted sequences in the hyperacute/acute phase (i.e. within three days from thrombus formation) [43]. In the acute phase the use of DWI can increase intramural thrombus visualization [44, 45] (Fig. 8).

MRA may show lumen narrowing due to expansion of an intramural hematoma, but also dilation of the arterial diameter in case of subadventitial dissection with formation of a pseudoaneurysm.

Vessel wall MR (VW-MR) imaging can complement findings of conventional lumen imaging (CTA, MRA and DSA) in the visualization of arterial dissection. VW-MR imaging is generally based on 3D volumetric sequences with fat suppression and optimized blood flow suppression [19]. Despite the improvement of the diagnostic accuracy of arterial dissection, VW-MR remains a heterogeneous technique in terms of sequence standardization, with a future need for better optimization across institutions [46]. In general, the frequency of patients with absolute contraindications to MRI is reported to be about 0.41%, among which is particularly relevant in trauma a 0,23% of patients harboring shrapnels in sensitive anatomical locations [47]. Moreover, in hemodynamically unstable trauma patients, transportation to the MRI suite, complexities related to monitoring in the MR environment and the overall length of images acquisition are a limit to the use of MR imaging.

#### Post-traumatic aneurysms

**Fig. 9:**
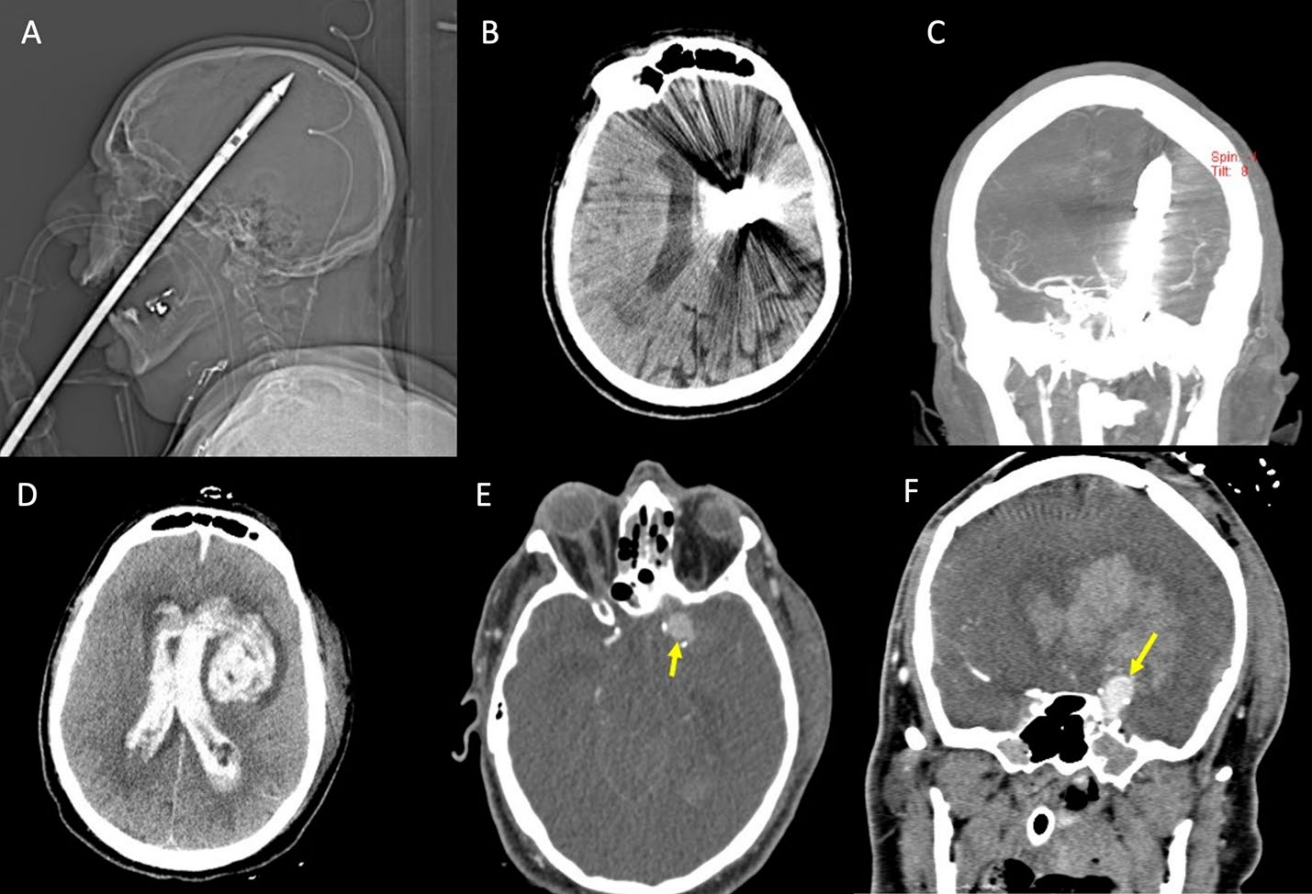
Patient in the sixth decade of life with a penetrating spear gun harpoon trauma (**A**, scout image). The harpoon penetrated the brain through the hard palate and the cranial base, with absence of significant cerebral hemorrhagic hyperdensity on non-enhanced CT (**B**), and a possible slight narrowing of the left distal middle cerebral artery on CTA (**C**), in the presence of metal artifact. Bottom row shows the post-surgical control, after removal of the harpoon, demonstrating the occurrence of ventricular hemorrhage and left intraparenchymal hematoma on non-enhanced CT (**D**), with evidence of a pseudoaneurysm arising from the supraclinoid left internal carotid artery on CTA (**E **and **F**, arrows)

Traumatic intracranial aneurysms are rare, as they represent less than 1% of all cerebral aneurysms, and are more common in the pediatric population [43, 48]. Traumatic aneurysms have been more often reported following blunt trauma. In the setting of penetrating trauma, stab wounds more frequently cause traumatic aneurysms (Fig. 9).

Delayed intracranial hemorrhage is the most common clinical manifestation with an average time of presentation from trauma of approximately 21 days. Bleeding can occur in the subarachnoid space, intraparenchymal, intraventricular or subdural and have a reported mortality rate as high as 50%. A different, dangerous presentation is massive epistaxis, which occurs when the aneurysm ruptures into the sphenoid sinus [3].

A histological categorization of traumatic aneurysms includes false, true or mixed lesions. False aneurysms are the most common. They are characterized by the disruption of all three layers of the vessel wall, surrounded by a contained hematoma; the development of a false lumen creates the aneurysmal dilatation [48]. True aneurysms are characterized by an intact adventitia with disruption of the intima and variable involvement of the internal elastic layer and of the tunica media. Mixed aneurysms start as true aneurysms but they then undergo rupture with a containing hematoma with false lumen formation. This classification has little clinical implication. Larson [48] classified traumatic aneurysms in proximal (vertebrobasilar, infraclinoid carotid artery and supraclinoid carotid artery aneurysms) and distal to the circle of Willis (subcortical and cortical aneurysms), an anatomical classification that can reflect different mechanisms of aneurysm formation.

Infraclinoid and basilar aneurysms are commonly related to skull base fractures, while in the supraclinoid segment mechanisms include impact with anterior clinoid process or stretching of this relatively more mobile segment of the artery.

The formation of subcortical and cortical aneurysms can be related to the impact of the vessel against the relatively rigid dural reflections (falx cerebri and tentorium) and to depressed skull fractures [49].

Clinical presentation also varies with anatomical location, in particular infraclinoid carotid artery aneurysms can present with cranial nerve palsies, carotid cavernous fistula (CCF) or massive epistaxis. However, clinical management does not necessarily follow the current Guidelines for aneurysmal SAH (aSAH) [50], despite blood pressure management and hemodynamic management to minimize bleeding are key factors in this context. The clinical management of traumatic SAH differs from the aSAH, as the pathophysiology, the risk of vasospasm and delayed cerebral ischemia are different. For instance, blood pressure management and targets, prophylactic use of nimodipine are not well established in traumatic SAH.

Non-contrast CT of post-traumatic aneurysms may show a hyperdense paravascular hematoma, but CTA is the imaging method of choice for the diagnosis and should be performed according to the screening criteria for blunt and penetrating cerebro-vascular injury. Suspicious findings should be further evaluated by DSA to confirm diagnosis and plan prompt endovascular treatment [51].

#### Post-traumatic artero-venous fistulas

Post-traumatic artero-venous fistulas (AVF) are acquired abnormal communications between an artery and a neighboring venous space, with blood shunting from higher pressure arterial compartment to lower pressure venous system [52]. In patients with predisposing factors, such as Ehler Danlos syndrome or other connective tissue disorders, even a minor trauma can lead to damage of the arterial wall and AV shunt formation [53].

As already mentioned, post-traumatic AVF are high flow shunts and they can manifest with several neurological symptoms depending on the pattern of venous drainage as well as the location of fistula. Most common presentation, such as intracranial hemorrhage and cranial nerves palsy are caused by venous congestion and compression of adjacent nervous structures by venous pouches with acute or delayed onset from days to weeks after trauma [54, 55].

**Fig. 10:**
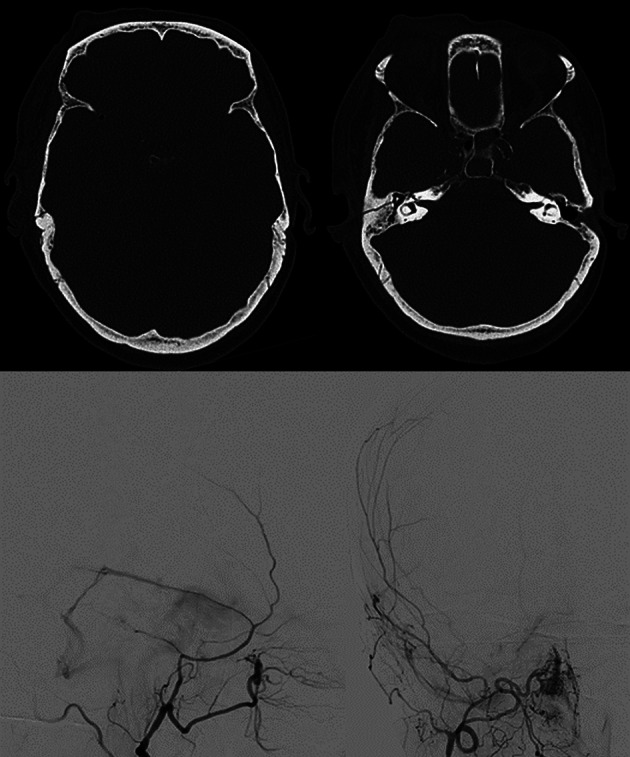
Blunt trauma in a patient in the early eighth decade of life. CT scan (top row) shows a non-otic longitudinal fracture of the right petrous bone. DSA (bottom row) performed while injecting the right external carotid artery territories, demonstrates the presence of a post-traumatic dural AVF between the posterior branch of the middle meningeal artery and the ipsilateral transverse sinus (Borden type I)

Carotid cavernous-fistula (CCF) is the most common type of post-traumatic artero-venous fistula, encountered in 0.2% of patients with craniocerebral trauma and in up to 4% of patients with skull base fractures [56], followed by vertebro-vertebral fistula (less than 10% of traumatic fistula) and fistulas from branches of external carotid artery, most commonly from middle meningeal and occipital arteries in proximity to skull fractures [56, 57] (Fig. 10).

#### Carotid cavernous-fistula

Carotid cavernous-fistula (CCF) is defined by an abnormal shunt between the internal carotid artery and the cavernous sinus. CCF can develop acutely, due to a direct damage to the walls of the intracavernous portion of the internal carotid artery (ICA), or may appear after a temporal delay from trauma, following the development and rupture of a post-traumatic aneurysm [58].

CCFs are classified on the basis of their arterial supply as direct CCF (type A), or indirect CCF (type B-D), according to Barrow classification. Type A are direct shunts between the ICA and the cavernous sinus (CS), while type B, C and D are connections between the dural branches of ICA, dural branches of external carotid artery (ECA), or branches from both external and internal carotid artery and CS respectively.

**Fig. 11:**
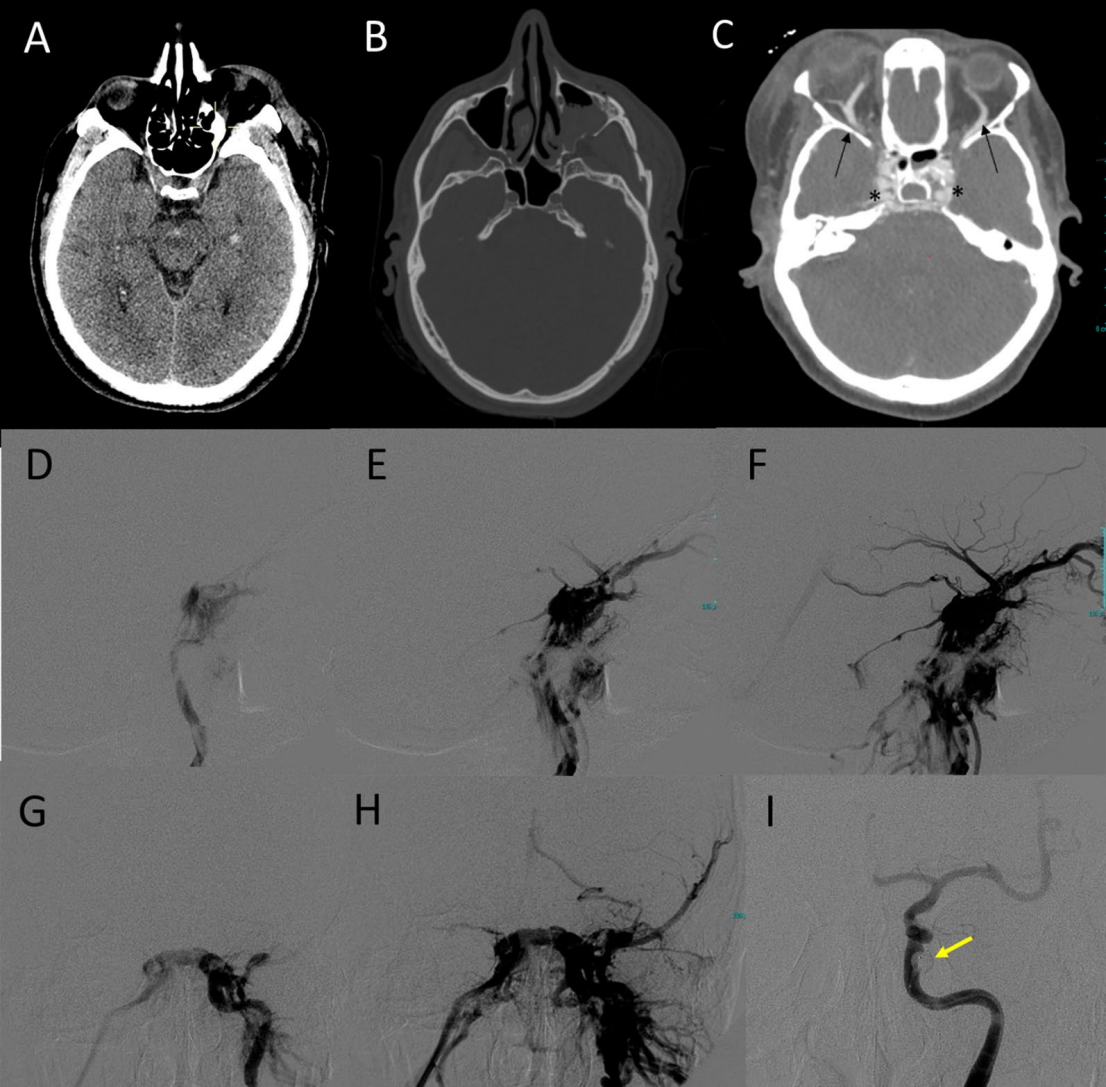
Post-traumatic direct CCF of a patient in the late sixth decade of life following a car accident. Non-enhanced CT scan (**A**, **B**) shows the presence of minimal subarachnoid hemorrhage in interpeduncular cistern and a small temporo-mesial intraparenchymal hemorrhage (**A**), with multiple fractures involving the left zygoma, the left maxillary sinus (**B**) and the left mandibular angle (not shown). CTA (**C**) shows prominent opacification and bulging of the cavernous sinus on both sides (asterisks) and bilateral enlargement of the superior ophtalmic veins (arrows). DSA (middle and bottom row) was performed two days later and showed a left CCF (**D**, **E**, **F**: lateral view; **G**, **H**: frontal view) with evidence of venous drainage via the anterior route, via the postero-inferior route and with cortical drainage through the left spheno-parietal sinus. Occlusion of the CCF was obtained with a detachable balloon (**I**, yellow arrow)

Direct CCF (type A) is the most common traumatic fistula, with a high flow shunt (Fig. 11).

The clinical picture is variable, and this variability depends on shunt velocity and the pressure within the venous structures and, most importantly, on the type of venous drainage, which affects the prognosis. Thus, classifications taking into account the type of venous drainage can be valuable to predict complications and to decide the treatment approach.

**Fig. 12:**
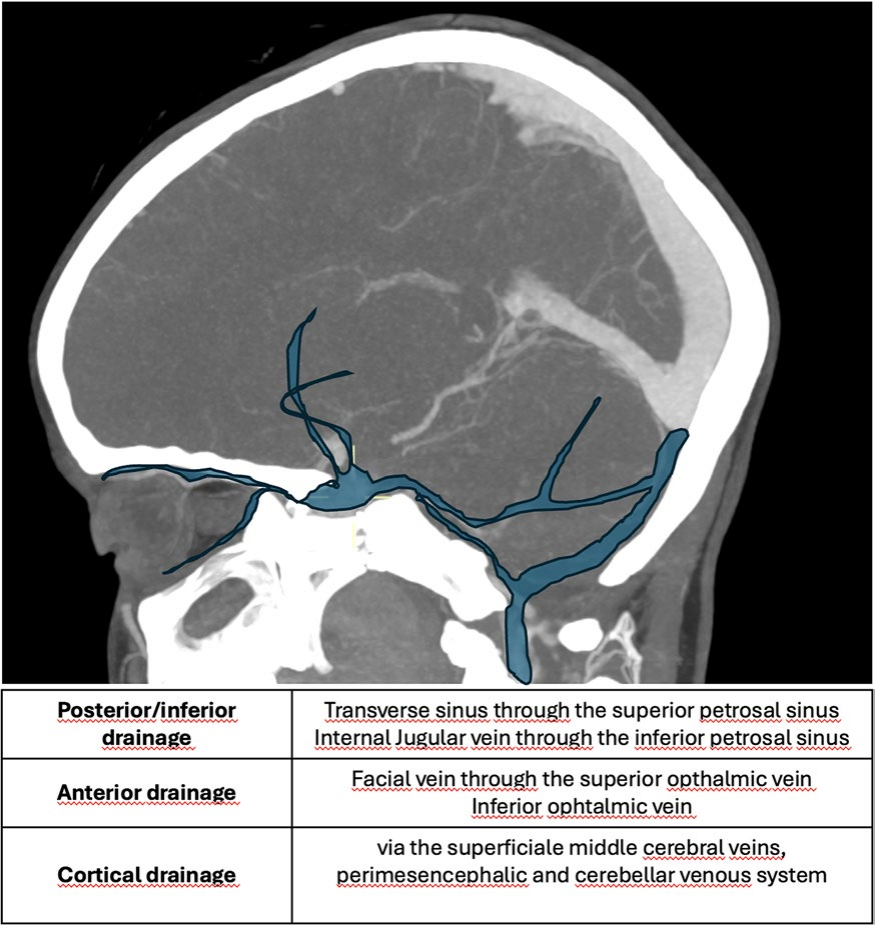
Schematic representation of the types of venous drainage

According to Thomas et al. [58, 59], CCFs venous drainage can follow three different routes: posterior/inferior drainage only (mainly via the superior and inferior petrosal sinuses), anterior drainage (through the superior and inferior ophthalmic veins) and cortical drainage (via the superficial middle cerebral veins and the perimesencephalic and cerebellar venous systems) (Fig. 12) [59, 60].

CCF can be classified, according to the different drainage, in 5 different types (Table 4).

**Table 4:** Cavernous carotid fistula (CCF) classification based on venous drainage

CCF classification system based on venous drainage pattern	
Type	Venous drainage
**1**	Posterior/inferior drainage only
**2**	Posterior/inferior and anterior drainage
**3**	Anterior drainage only
**4**	Retrograde drainage into cortical veins +/- other routes of venous drainage
**5**	High-flow direct shunt between cavernous internal carotid artery and cavernous sinus (Barrow type A) +/- multiple routes of venous drainage

The anterior route is the most common and it is usually related to orbital symptoms, including proptosis, due to increased pressure in the veins around the eye, conjunctiva hyperemia, due to arterialization of conjunctival and episcleral veins, ophthalmoplegia and diplopia, glaucoma, ocular/periorbital pain, retinal hemorrhage with visual loss and vascular bruit, caused by turbulent blood flow in the fistula [61].

The congestion of the cavernous sinus may result in pulsating ophthalmoplegia, diplopia, ptosis and anisocoria. Strabismus is one of the most common and earliest symptoms due to the susceptibility of the VI cranial nerve to increased pressure within the cavernous sinus [61].

The cortical and posterior routes are the most dangerous because they can cause hemorrhage following venous congestion, seizures, and focal neurological deficits. Posterior drainage can also rarely lead to myelopathy in case of perimedullary veins outflow via the petrosal sinus system [62].

An extremely rare but potentially life-threatening clinical presentation is massive epistaxis, which occurs when there is communication between the CCF and the sphenoid sinus through a discontinuous bone [58]. Epistaxis may take place acutely as a direct consequence of trauma or after the rupture of a post-traumatic aneurysm of the intracavernous ICA.

DSA represents the gold standard for evaluating CCFs and for the treatment. Nevertheless, in the suspicion of CCF, CT and CTA can establish the diagnosis of CCF before treatment and add relevant information. In CCF with anterior drainage through the ophthalmic veins, CT based imaging can show peculiar intraorbital findings such as unilateral or bilateral exophthalmos, dilatation and early enhancement of the superior ophthalmic vein (SOV), with extraocular muscle thickening and periorbital fat edema.

Other findings in CCF are unilateral bulging of a cavernous sinus, asymmetric enhancement and arterialization of the surrounding venous structure, engorgement of the venous sinuses and venous pouches formation [63]. In rare cases, intracranial hemorrhages or venous ischemia, subsequent to intracranial venous congestion, can be detected.

Similarly to CT, MRI can detect abnormal contours of the cavernous sinus, prominent flow voids and early enhancement [64]. Rent size and location of the shunt, draining veins and collateral communicating arteries can be seen as signal arterialization inside venous structure on MRI-Angiography with three dimensional time-of-flight, a technique that is very sensitive, but has low specificity [65–67]. Moreover, with arterial spin labeling (ASL), early shunting of magnetically labeled arterial blood can be seen as a hyperintense signal at the A-V shunt location [68, 69]. Susceptibility Weighted Imaging (SWI) can detect arterialized flow in superior ophthalmic vein (SOV) depicting changes in signal intensity related to the blood oxygenation; moreover SWI is less affected by acquisition artifacts (such as inclination of saturation band) than TOF and could help in differentiate between low and high flow fistulas [69].

#### Vertebral AVF (VAVF)

Artero-venous fistulas involving the vertebral arteries are less common than CCF. In VAVF a pathological direct shunt exists between the vertebral artery and the veins of the vertebral plexus. Knife trauma, gunshot wound or blunt trauma can cause VAVF.

**Fig. 13:**
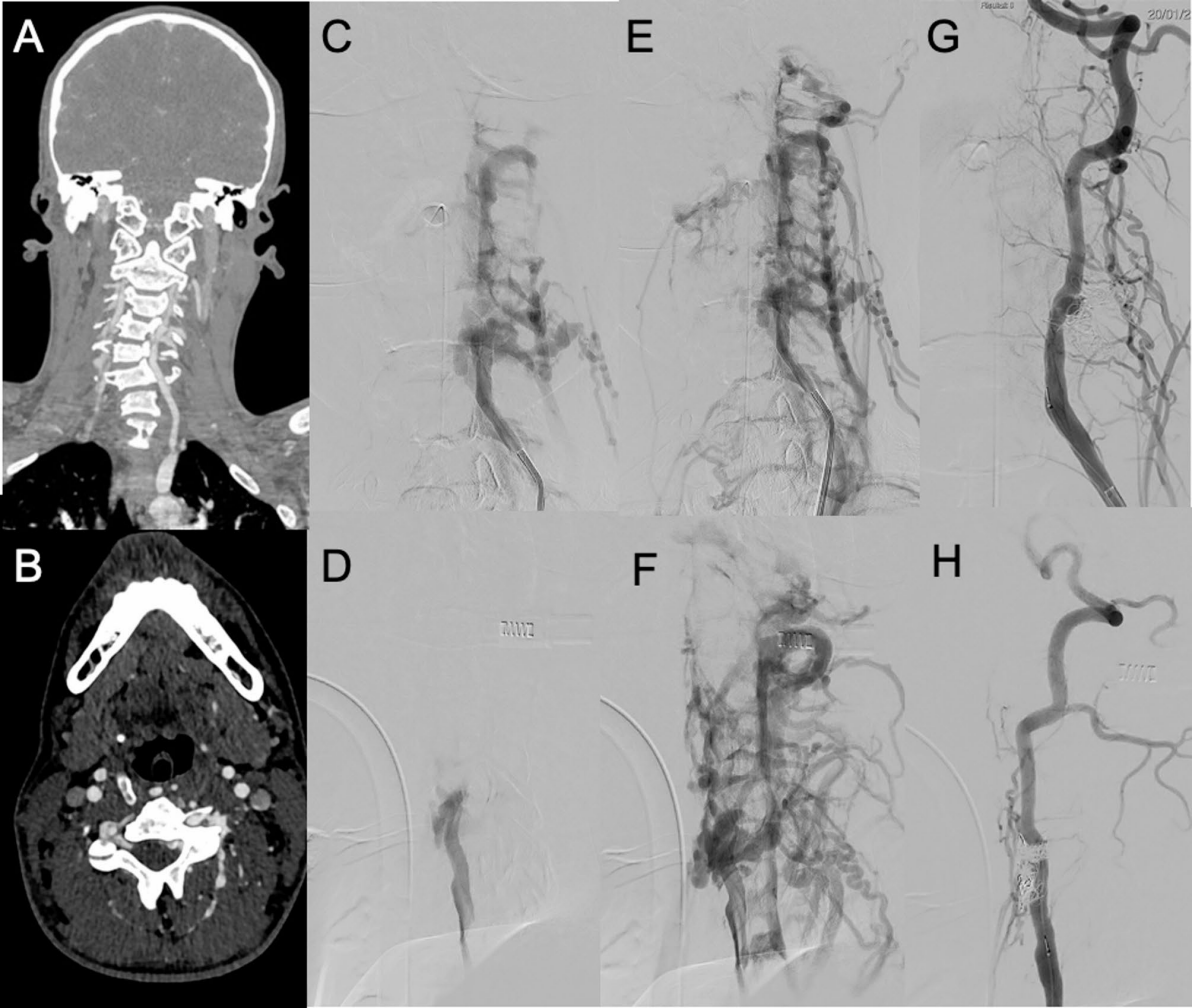
VAVF of an adolescent patient with neurofibromatosis 1 after a minor trauma. In **A **(coronal reconstructions) and **B **(axial native images) CTA shows asymmetric opacification of the cervical venous plexus, with early enhancement in the left side. In **C**, **D**, **E **and **F **the pre-operative DSA, performed while injecting the left vertebral artery, better demonstrates the presence of a VAVF between the left vertebral artery and the left cervical venous plexus, with evidence of dilated and tortuous arterialized veins (**C**, **E**: frontal views. **D**, **F**: lateral views). In **G **(frontal view) and **H **(lateral view) post-treatment results are shown, with complete occlusion of the fistula by coiling and preservation of the parent artery patency in the arterial phase

While spontaneous VAVF are more commonly located at C1-C2 and iatrogenic VAVF at C5 level, VAVF can occur throughout the cervical spine without a specific level [70] (Fig. 13).

Nevertheless, in penetrating trauma involving zone II of the neck (between the cricoid and the angle of the mandible) there can be concern for VAVF in the V2 segment, between C2 and C5 cervical vertebrae [71, 72].

Typically, VAVFs present with pulsatile tinnitus, bruit, vertigo, neck pain. Less common manifestations include subarachnoid hemorrhage, compressive myelo-radiculopathy, stealing phenomena (posterior circulation transient ischemic attacks, lower cranial nerves deficits) and reflux in perimedullary veins with risk of myelopathy and intramedullary hemorrhage.

CT-Angiography can reveal engorged epidural and paraspinal venous structures and extravascular contrast accumulation near the vertebral artery [71, 73].

MR-Angiography can show arterialized flow in the venous structures surrounding the vertebral arteries [60].

DSA represents the gold standard in the diagnostic phase to confirm clinical suspicion and define angioarchitecture of the VAVF. Moreover, the treatment of choice is endovascular and its indications are based on the presence of clinical symptoms, and in VAVF with retrograde, intracranial, or spinal cord venous drainage [70].

#### Venous thrombosis

Traumatic brain injury is an uncommon cause of dural venous sinus thrombosis and can be related to skull fractures extending to dural sinuses or jugular bulbs predisposing to intraluminal thrombus formation or to extrinsic compression of the dural sinuses with bone fragments or hematomas limiting venous flow [74]. Due to the complexity of its presentation, dural venous sinus thrombosis has been probably in the past under-diagnosed in this specific setting, especially in the early, acute phase. Nevertheless, with the advent and wide availability of CTA, the diagnosis has become easier and thus the frequency of post-traumatic venous thrombosis has increased. A recent review and meta-analysis found a prevalence of 26.2% of dural venous sinus thrombosis in patients with skull fractures adjacent to a venous sinus [75]. In large case series [76, 77] post-traumatic venous thrombosis was always associated with skull fractures involving the sinuses. Delgado Almandoz et al., by using MDCT venography, depicted thrombosis in 40.7% of the fractures extending to dural sinuses or jugular bulbs.

**Fig. 14:**
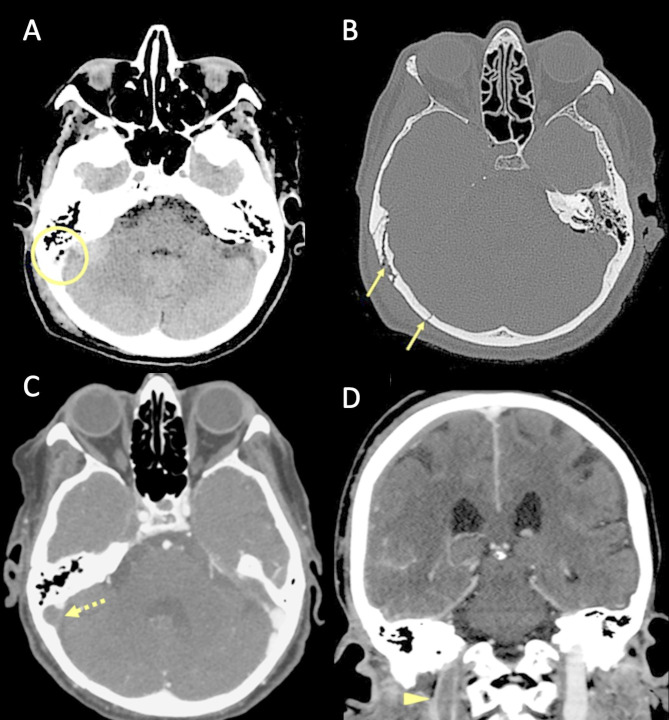
Blunt trauma in a patient at the beginning of the ninth decade of life. Non-enhanced CT scan (**A**, **B**) obtained at presentation shows the presence of air bubbles and hyperdensity of the right transverse sinus (**A**, circle), adjacent to a diastasis of the occipito-mastoid suture, with a fracture of the ipsilateral squamous occipital bone (**B**, arrows). Follow-up CTA performed after 48 h (**C**, **D**), shows a lack of opacification of the right transverse sinus (**C**, dotted arrow) and of the ipsilateral jugular vein (**D**, arrowhead), consistent with venous thrombosis

**Fig. 15:**
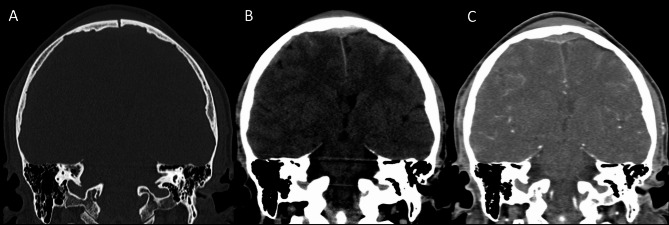
Blunt trauma in a patient in their twenties. Non-enhanced brain CT shows a post-traumatic fracture of the frontal bone (**A**), with evidence of an extra-axial blood collection, consistent with midline epidural hematoma of the cranial vault (**B**). CTV (**C**) demonstrates lack of opacification of the superior sagittal sinus, resulting from compression by the epidural hematoma

Several pathophysiological mechanisms may be associated with traumatic cerebral venous thrombosis. Damage to the venous sinus dura mater adjacent to a skull fracture may disrupt the endothelial integrity and trauma associated endothelial cell surface network damage contribute to coagulopathy [78] (Fig. 14). Another mechanism associated with CVT in the setting of head trauma is extrinsic dural venous sinus compression [75]. Sinus compression can be secondary to displaced fracture fragments, extra-axial haematoma (Fig. 15) or pneumocephalus adjacent to the dural venous sinus.

**Fig. 16:**
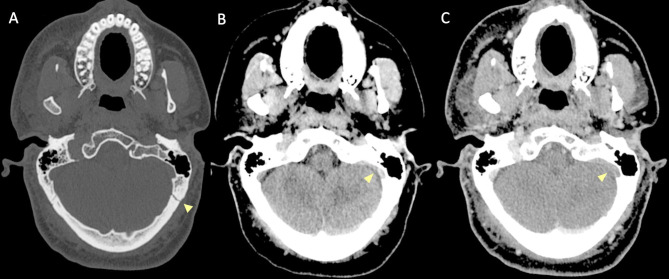
CT scan of a young adult patient involved in a motor vehicle accident. A post-traumatic diastasis of the left occipito-mastoid suture is shown (**A**, arrowhead) with hyperdensity of the left sigmoid sinus (**B**, arrowhead). A fracture of the left anterior occipital bone, posterior to the ipsilateral hypoglossal canal is also shown (**A**). The CTV (**C**) shows lack of opacification of the left sigmoid sinus, consistent with venous thrombosis (arrowhead)

Petrous temporal fractures can affect the transverse sinus, sigmoid sinus and the jugular bulb, whereas frontal, parietal or occipital fractures can affect the superior sagittal sinus (SSS). In the series reported by Delgado Almandoz, the fractures of the petrous temporal bone extending to the transverse sinus, sigmoid sinus or jugular bulb had a higher risk (50%) of traumatic CVT with respect to occipital bone fractures (34%). Occipital bone fractures extending to the SSS had a higher risk of CVT (67%) than parietal bones (39%) or frontal bones (24%) [77]. Skull sutures’ diastasis may also be associated with venous sinus thrombosis (Fig. 16). Patients with blunt head trauma, without fractures adjacent to the dural sinuses or jugular bulb and without extrinsic compression of a sinus are less likely to develop CVT in the setting of trauma [79].

**Fig. 17:**
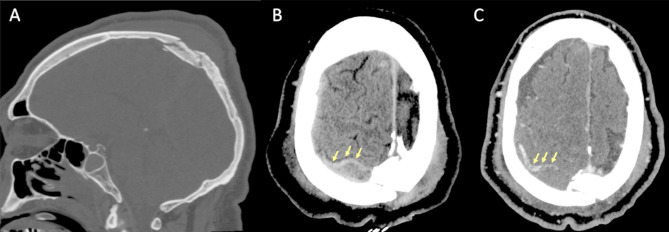
CT scan of a patient in the early sixth decade of life with a depressed skull fracture of the parietal bone (**A**), with findings consistent with thrombosis of a cortical vein. Non-contrast CT scan (**B**) shows a linear hyperdensity at the level of the right post-central sulcus (yellow arrows) along the presumed anatomical course of a cortical vein, adjacent to the fracture. Follow-up CTV at 48 h (**C**), shows absent/irregular opacification along the course of a right parietal cortical vein (arrows), corresponding to the non-contrast finding, consistent with cortical vein thrombosis. The superior sagittal sinus (not shown) was patent

On non-enhanced CT scan, hyperdensity of a dural venous sinus or along the anatomical course of a cortical vein might be the primary sign of an underlying thrombosis. In major traumas, concomitant presence of hyperdense hemorrhage adjacent to a thrombosed venous structure, might decrease the conspicuity of this sign and the venous thrombosis could be overlooked. The presence of air bubbles in the venous system adjacent to a skull fracture might represent a red flag to suspect venous thrombosis which should be confirmed with a CT Venography (CTV) (Fig. 14) [79]. Air in the cavernous can be related to trauma [80] but also in more benign conditions such as through phleboclysis injection [81]. A post-traumatic thrombosis involving a cortical vein should be suspected in the presence of a depressed skull fracture with evidence of a linear hyperdensity on non-enhanced CT along the presumed anatomical course of the vein (Fig. 17).

CTV enables a rapid assessment of the deep and superficial cerebral venous system and is the modality of choice for the evaluation of post traumatic cerebral venous thrombosis. It depicts vascular enhancement in the patent venous structures and filling defects are the most frequent signs of venous thrombosis. In studies assessing the diagnostic performance of CTV in non-traumatic cerebral venous thrombosis, sensitivities and specificities range from 75 to 100% depending also on the location of the thrombosis.

CTV can be easily added to the diagnostic protocol in trauma patients when a cerebral venous thrombosis is suspected. Its advantages over MRI include fast acquisition times, wide availability and less contraindications including the possibility to acquire images in patients with pacemakers, ferromagnetic devices and in the setting of acute trauma in patients with concern of foreign metal objects including shrapnels from trauma itself.

Small studies comparing CTV and DSA in depicting venous anatomy and pathology have demonstrated similar diagnostic performances of the two modalities [82]. Angiography is today seldom used and typically reserved to cases which are selected for endovascular treatment or in the rare cases that remains with equivocal findings after CT/CTV and or MR/MRV imaging studies. DSA findings includes failure of sinus opacification, venous congestion with dilated veins, enlarged collateral drainage pathways or flow reversal.

MRI is less commonly used in the acute setting in trauma. In general, interpretation of MRI findings requires knowledge of signal intensities changes as the thrombus ages. Conventional MRI is more sensitive than UHCT in the detection of thrombus and loss of regular flow voids should lead to investigation for CVT. In the early phases (within approximately 5 days from thrombus formation) deoxyhemoglobin within the thrombus is reflected by isointensity on T1 weighted images and hypo-intensity on T2 weighted images, thus mimicking a regular flow void; SWI and GRE T2 sequences may show blooming at the location of the thrombosed sinus. In this stage a contrast enhanced MRV study can be essential to avoid diagnostic mistakes. In the subacute stage (6 to 15 days) thrombus is hyperintense on T1 and T2 weighted images reflecting the presence of methaemoglobin, a finding which is less equivocal. MRI can better characterize the possible consequences of venous thrombosis in the brain tissue such as vasogenic edema, venous infarction and hemorrhagic transformation [83].

Management of nontraumatic CVT with anticoagulation is well established, but optimal treatment of traumatic CVT is still controversial [84]. In a recent series treatment of traumatic CVT included anticoagulation or antiplatelet while a conservative approach was utilized in up to 51% of patients. Active treatments were associated with more bleeding complications than conservative treatment [85]. According to Hersch et al. [86], 22/38 patients with traumatic CVT were treated with anticoagulation with resolution of thrombosis in 50% of cases at three months; among the treated patients there were minor complications in 14% of cases (including gastrointestinal bleeding), while another 14% demonstrated an increase of intracranial hemorrhage, with mortality in 4.5%. Antosson et al. [87] in 32 patients with traumatic CVT treated with heparin infusion of with low molecular weight heparin did not observe hemorrhagic complications. When anticoagulation is precluded mechanical endovascular thrombectomy might be considered as an option [88, 89].

## Conclusions

Post-traumatic neurovascular complications might lead to severe consequences and be life threatening. Early diagnosis is crucial to optimize patient management and improve clinical outcomes. Patients may present with multiorgan damage or injuries, which limit the decision making unless the hemorrhagic risks are well controlled.

Hence, the clinicians must not overlook signs suggestive of underlying neurovascular complications and should be trained to recognize the instances which require dedicated neurovascular imaging protocols. The correct interpretation of neurovascular imaging in TBI might be facilitated by artificial intelligence tools, already utilized in patients with non-traumatic neurovascular pathology [90, 91]. Further directions in the field could include the use of information obtained with neuroimaging in improving the prediction of prognosis in TBI patients with neurovascular lesions [92].

## Data Availability

No datasets were generated or analysed during the current study.

## Abbreviations

TBI: traumatic brain injury
CCF: carotid-cavernous fistula
CT: Computed Tomography
CTA: Computed Tomography Angiography
MRI: Magnetic resonance imaging
DSA: Digital Subtraction Angiography
TBI: Traumatic Brain Injury
AVF: Artero-Venous Fistula
BCVI: Blunt Cerebro-Vascular Injury
MRA: Magnetic Resonance Angiography
CTV: Computed Tomography Venography
MRV: Magnetic Resonance Venography
